# Assessing viability of a minimally invasive autopsy technique in ascertaining the probable cause of death in patients who were SARS CoV19 positive at the time of their demise

**DOI:** 10.1186/s42047-021-00094-3

**Published:** 2021-07-13

**Authors:** Ajay H. Bhandarwar, Girish D. Bakhshi, Eham Arora, Nikhil Dhimole, Sanjay R. Bijwe, Shubhangi V. Agale, Megha S. Kinake, Shilpa Domkundwar, Yogesh Thube

**Affiliations:** 1 Department of General Surgery, Grant Medical College and Sir JJ Group of Hospitals, Mumbai, India; 2 Department of Pathology, Grant Medical College and Sir JJ Group of Hospitals, Byculla, Mumbai, 400008 India; 3 Department of Radiodiagnosis, Grant Medical College and Sir JJ Group of Hospitals, Mumbai, India

**Keywords:** Minimally invasive autopsy, Covid-19, Autopsy, Histopathology

## Abstract

**Background:**

SARS CoV-19 was declared as a pandemic by the World Health Organization (WHO), raising up challenges on various levels ranging from therapeutics to diagnostics. The conventional autopsy technique may pose a health hazard to health care workers. A minimally invasive autopsy technique can diminish this hazard.

**Materials and methods:**

Between August and November 2020, 51 patients who were suffering from Covid-19 at the time of their demise were included. A novel minimally invasive ultrasound-guided technique for procuring tissue samples of major organs was employed which were thereafter subject to histopathological examination. A detailed review of the course in hospital was noted. An analysis was performed to correlate the cause of death ascertained from our minimally invasive technique with the cause of death ascertained clinically.

**Results:**

There was adequate tissue sampling in 45 cases, where the minimally invasive autopsy technique confirmed the cause of death in all 45 cases (100%) and made it more specific in 5 cases (11.11%).

**Conclusion:**

Minimally Invasive Autopsy is an easily reproducible technique which has the potential to strengthen the probable the cause of death with reasonable certainty while ensuring safety and ethics.

## Background

The outbreak of SARS CoV19 infection has spread all over the world and it was declared as an emergency on 11th March 2020 by the World Health Organisation (WHO, 2020). Being a novel virus, little was known about it and the constellation of pathologies it caused. The initial lack of information hindered goal directed therapeutics, and therefore a more focused evaluation in the cytopathological changes of the major organ systems was warranted. Due to the vague and wide spectrum of symptoms caused by the disease, as well its overlap and increased prevalence in patients with multiple comorbidities, there can often be a dilemma in certifying the cause of death. The conventional methods of post-mortem examination and procurement of tissue samples is time and resource-intensive, in addition to the apprehension about transmission of virions from the cadavers to the examiners.

**Fig. 1:**
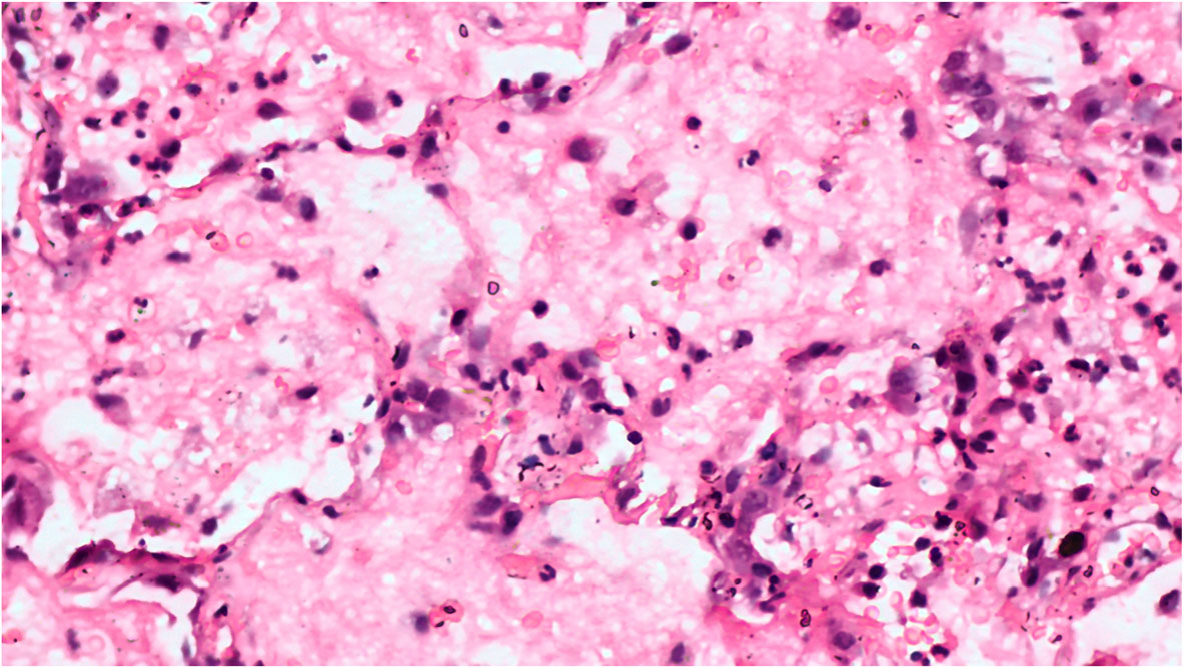
Microphotograph of pulmonary tissue showing early change of diffuse alveolar damage (H&E, X200)

**Fig. 2:**
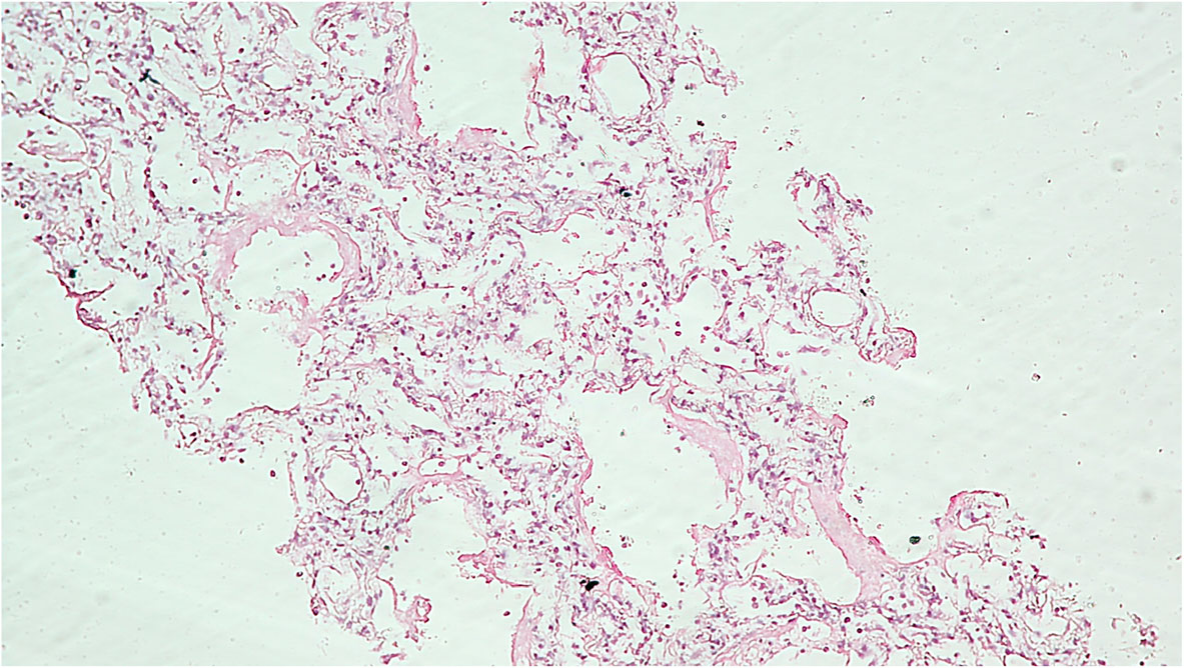
Microphotograph of pulmonary tissue showing hyaline membrane formation with interstitial widening and inflammation (H&E, X400)

**Fig. 3:**
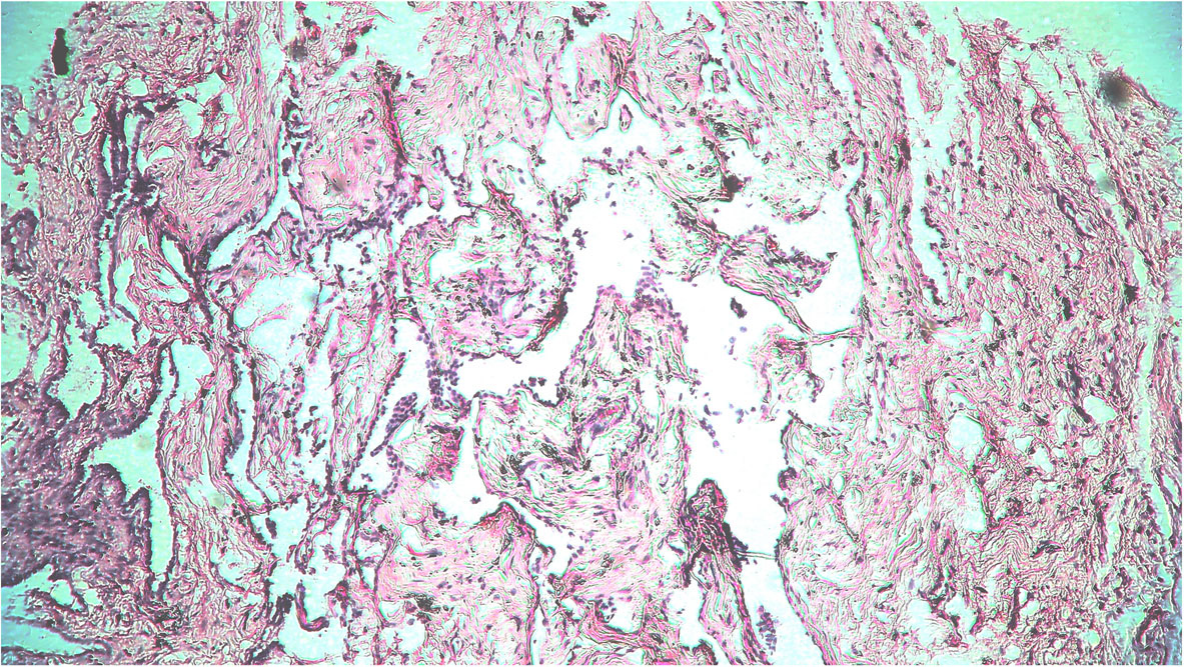
Microphotograph of pulmonary tissue showing fibrotic stage of diffuse alveolar damage (H&E, X400)

**Fig. 4:**
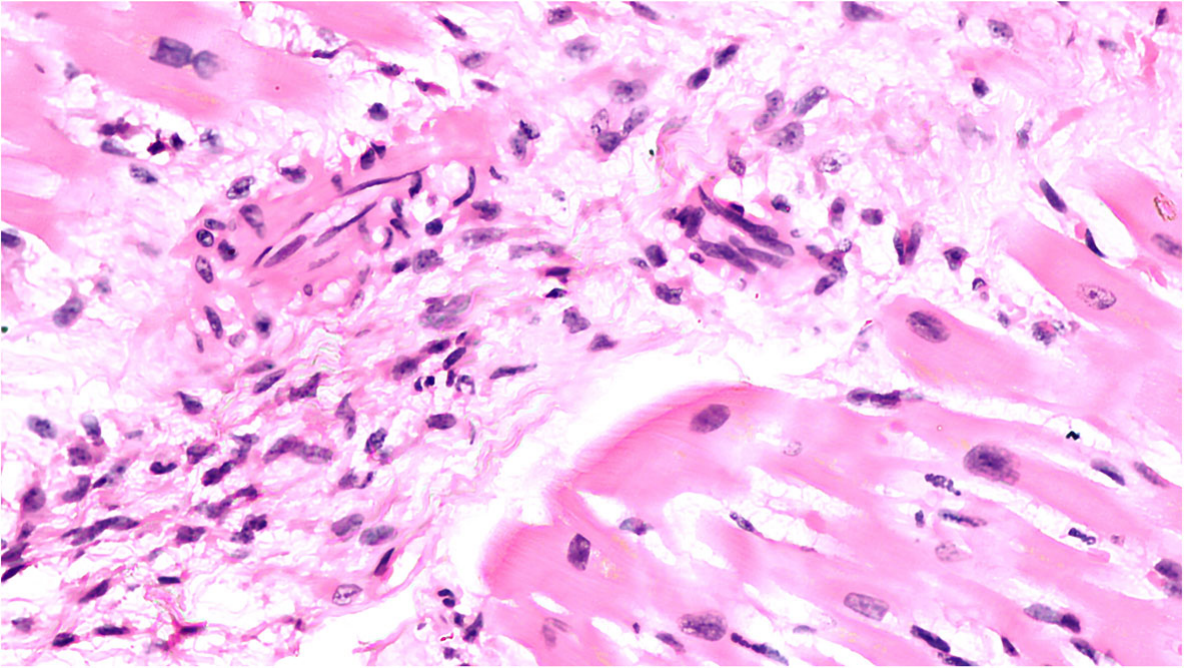
Microphotograph of myocardial tissue showing myocarditis. There is an inflammatory infiltrate involving myocardial fibres with myocytolysis (H&E, X400)

**Fig. 5:**
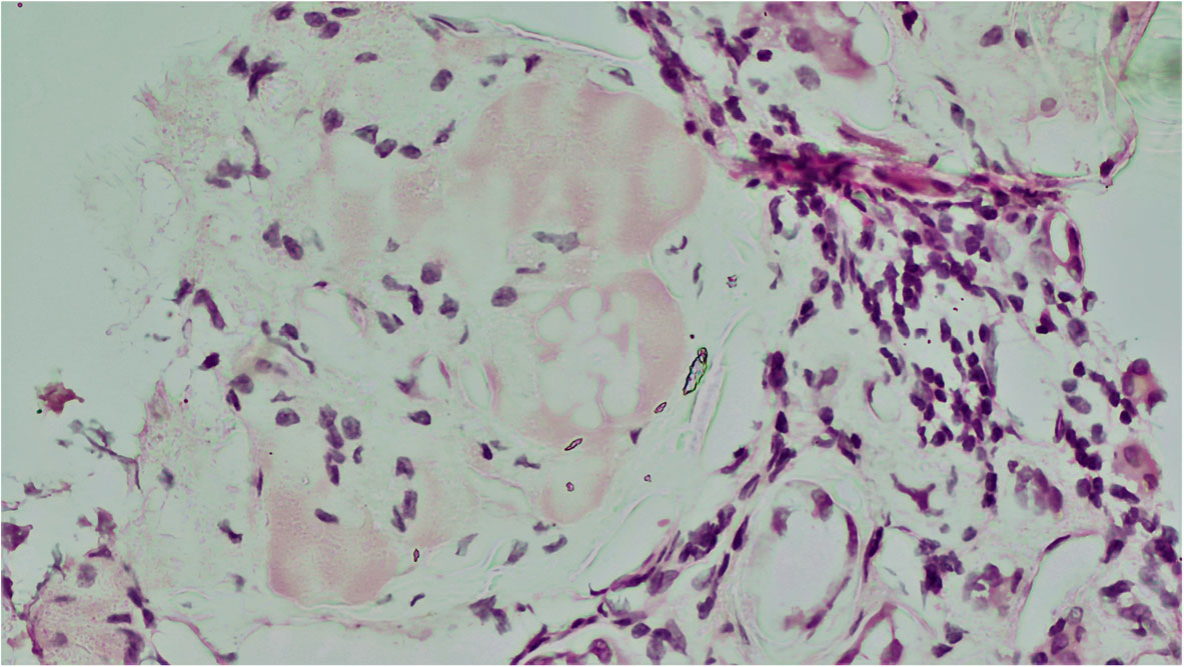
Renal microphotograph showing ovoid, eosinophilic nodules in mesangium characteristic of diabetic nephropathy(H&E, X400)

**Table 1:** Patient demographics, clinical parameters and cause of death noted in our MIA series

Total number of patients: 51	N (%)
Age (years)	53.7 (16–78)
< 10 years	0
10–20 years	1 (1.96)
20–30 years	2 (3.92)
30–40 years	5 (9.80)
40–50 years	10 (19.60)
50–60 years	14 (27.45)
60–70 years	13 (25.49)
> 70 years	6 (11.76)
Sex	
Male	29 (56.86)
Female	22 (43.13)
Comorbidities	
Diabetes mellitus	32 (62.74)
Hypertension	19 (37.25)
Bronchial asthma	5 (9.80)
Ischemic heart disease	11 (21.56)
Chronic kidney disease	7 (13.72)
Chronic liver disease	2 (3.92)
Need for invasive ventilation	
Yes	44 (86.27)
No	7 (13.72)
Clinical cause of death	
Type 1 Respiratory failure	19 (37.25)
Type 2 Respiratory failure	2 (3.92)
Septic shock	8 (15.68)
Intracranial bleed	2 (3.92)
Myocardial infarction	3 (5.88)
Viral meningitis	3 (5.88)
Lobar pneumonia	9 (17.64)
Acute on chronic renal failure	4 (7.84)
Hepatic encephalopathy	1 (1.96)

**Table 2:** Findings in microscopic analysis of MIA tissue samples

Histopathological finding	N (%)
Lung (49)	
Diffuse alveolar damage (DAD)	44 (89.79)
Early DAD	14 (28.57)
Proliferative or organising phase of DAD	20 (40.81)
Late DAD	10 (20.40)
Interstitial pneumonia	4 (8.16)
Lung abscess	1 (2.04)
Pulmonary oedema	6 (12.24)
Microthrombi	21 (42.85)
Hyaline membrane	19 (39.77)
Type II pneumocyte hyperplasia	15 (30.61)
Intra-alveolar hemorrhage	12 (24.48)
Myocardium (51)	
Focal myocardial hypertrophy	39 (79.59)
Interstitial myocarditis	2 (4.08)
Interstitial fibrosis	8 (16.32)
Plasma cell and neutrophilic infiltration	6 (12.24)
Kidney (47)	
Cloudy degeneration of tubules	24 (51.06)
Thyroidisation of tubules	7 (14.89)
Tubular atrophy	6 (12.76)
Glomerular congestion	18 (38.29)
Mesangial proliferation	12 (25.53)
Glomerulosclerosis	7 (14.89)
Interstitial inflammation	12 (25.53)
Hyaline arteriosclerosis	9 (19.14)
Liver (49)	
Hepatic sinusoidal dilatation and congestion	29 (59.18)
Vacuolar degeneration	22 (44.89)
Microvesicular fatty change	18 (36.3)
Cholestasis	5 (10.20)
Lymphocytic infiltration	30 (61.22)
Central venous congestion	14 (28.57)
Spleen (46)	
Sinusoidal congestion	22 (47.83)
Amyloidosis	1 (2.17)

**Table 3:** Findings of MIA and their influence on the clinical cause of death

Total number of patients sampled	Total number of patients with adequate sampling	Total number of cases where MIA aligned with the probable cause of death determined by treating physician teams	Total number of cases where MIA made the cause of death more specific
51	45	45 (100%)	5 (11.11%)

**Table 4:** Values of inflammatory markers (*n* = 51)

Laboratory parameter	Mean ± Standard deviation
Serum ferritin	608.3 ng/mL ± 32.5 ng/mL
Serum Interleukin-6	171.5 pg/mL ± 17.2 pg/mL
Serum D-dimer	1559.74 U/L ± 378 U/L
Serum Lactate Dehydrogenase (LDH)	5553.7 ng/mL ± 798.4 ng/mL

Obtaining tissue samples for histopathological examination using a minimally invasive technique can be a viable time and resource-saving technique (Castillo et al., 2015a). We describe the technique of minimally invasive autopsy (MIA) to obtain tissue samples in patients who were CoV19 SARS positive at the time of their demise. Furthermore, we studied the outcomes to ascertain the impact of the technique on adding to or modifying the certified cause of death.

## Materials and methods

This is a prospective observational study conducted at Grant Medical College and Sir JJ Group of Hospitals, Mumbai conducted between August and November 2020. The study was approved by the Institutional Ethics Committee. Fifty-one patients suffering from SARS CoV19 at the time of demise were enrolled in it after due consent from the next in kin.

Post mortem tissue sampling was performed using core-needle biopsy guns (Bard Davol Inc., USA) via a percutaneous route under ultrasound guidance. All tissue sample was performed within 2 h of cessation of cardiac activity. The site of tissue samples was cleaned, a 2 mm incision made with a knife and 4–6 specimen cores were obtained with the core-needle biopsy guns from each target organ – the lungs, myocardium, liver, kidneys, pancreas, preperitoneal fat and spleen. The tissue samples were preserved in a freshly prepared solution of 10% formaldehyde and dispatched for histopathological analysis. The slides obtained from paraffin-embedded blocks were stained by hematoxylin and eosin stain. Morphology of the organ tissue (lung, heart, kidney, liver, brain) submitted was studied and recorded by in-house pathologists having expertise in the particular organ system.

Demographic data, hematological, radiological and biochemical investigation results, clinical course in hospital prior to demise and medical cause of death as best ascertained by the treating physician teams were noted. An analysis was performed comparing the inferences from MIA samples with the clinically ascertained cause of death to study the correlation between the two. MIA samples were noted to either confirm and support, add on or contradict the clinical cause of death (CCOD).

## Results

Of the 51 deceased (Table 1) who were subjected to MIA, we found inadequate pulmonary tissue sampling in two cases. Out of the 49 satisfactory samples studied, 14 (28.57%) showed early diffuse alveolar damage (DAD) (Fig. 1), 20 (40.81%) demonstrated proliferative or organising phase of DAD. 10 patients (20.40%) had late DAD on their microscopy findings (Fig. 3). Histopathology of 4 patients (8.16%) revealed interstitial pneumonia while 1 patient (2.04%) had a lung abscess. Findings suggestive of pulmonary oedema were noted in 6 samples (12.24%) while scattered microthrombi were observed in 21 samples. Hyaline membrane was noted in 19 (39.77%) of the samples (Fig. 2), type II pneumocyte hyperplasia was observed in 15 (30.61%) samples while another 12 (24.48%) samples showed intra-alveolar haemorrhage.

Out of the 51 myocardial samples studied, 39 (79.59%) showed focal myocardial hypertrophy, 2 (4.08%) had interstitial myocarditis (Fig. 4) whereas the other 10 (20.40%) specimens were essentially unremarkable. Changes like interstitial fibrosis were seen in 8 (16.32%) patients while the results displayed plasma cell and neutrophilic infiltration in 6 (12.24%) specimens.

47 satisfactory samples of renal autopsy tissues were secured which revealed cloudy degeneration of tubules in 24 patients (51.06%), which was the most common finding followed by thyroidisation of tubules in 7 patients (14.89%) and atrophy in 6 patients (12.76%). The remaining patients had unremarkable renal tubular morphology. The most frequent finding in the glomeruli was congestion, which was present in 18 patients (38.29%), followed by mesangial proliferation (Fig. 5) in 12 patients (25.53%) and glomerulosclerosis in 7 patients (14.89%). Interstitial inflammation was noted in 12 cases (25.53%) and hyaline arteriosclerosis was noted in another 9 cases (19.14%).

A total of 49 samples of liver autopsy were procured using minimally invasive techniques. Hepatic sinusoidal dilatation and congestion was the most prevalent finding present in 29 cases (59.18%). Hepatocytes most frequently showed vacuolar alterations which was present in 22 patients (44.89%), followed by microvesicular fatty changes present in 18 patients (36.3%) and cholestasis present in 5 patients (10.20%). In the remaining 16 cases, hepatocytes had unremarkable histopathology. Portal triads showed dense lymphocytic infiltration in 9 patients (18.36%), moderate lymphocytic infiltration in 16 cases (32.65%) and mild infiltration in 5 cases (10.20%). In the remaining 19 (38.77%) cases, the portal triad was unremarkable. 14 (28.57%) patients demonstrated central venous congestion.

Forty-six satisfactory samples of splenic tissue were obtained out of which 22 showed sinusoidal congestion (47.83%) and 1 sampled demonstrated evidence of amyloidosis (2.17%) (Table 2). Fifty satisfactory samples of preperitoneal fat were secured, all of which were unremarkable.

A retrospective review of radio-imaging of the study subjects an average CT Severity Score of 17.3 out of a possible 25.

Of the 51 cases who were enrolled in the study, there was inadequate pulmonary tissue sampling in 6 cases and they were therefore excluded from the analysis. In the 45 valid samples, outcomes of the MIA procedure confirmed the cause of death in all the cases (Table 3). Also, it made the cause of death more specific in five cases. In four cases, the findings of renal autopsy specimens augmented the clinical suspicion of acute exacerbation of chronic renal disease. The acute renal dysfunction was evident in their biochemical investigations. In another case, the finding of steatohepatitis with chronic hepatic failure, helped to explain the unexpected early hepatic dysfunction and encephalopathy in one patient thus augmenting the clinical cause of death. The cases' biochemical inflammatory markers are summarized in Table 4.

Amongst the four patients with newly diagnosed renal pathologies, derangement of renal function tests was noted in all of them. A look into the values of inflammatory markers of these patients revealed significant elevation in all of them.

## Discussion

As a result of our research, we were able to gain insights into the histopathological changes in different organ systems as a result of the SARS -CoV19 infection. These findings included proliferative diffuse alveolar damage present in 40.81% of the patients which correlates well to other studies (Carsana et al., n.d.; Menter et al., 2020). Liver autopsy specimens in our study contrasted with the findings of Sonzogni A et al (Sonzogni A et al, 2020). who observed that the liver was not a primary target of Covid-19 infection and only vascular changes were noted. Most commonly findings like vacuolar degeneration and sinusoidal congestion and dilatation were noticed which did not corroborate with the patient’s clinical history and biochemistry before demise. It would therefore appear that these acute changes within the hepatic tissues were a result of the infection. However, we cannot rule out other confounding factors causing the same within our small cohort. Patterns in renal histopathology exhibited mild to severe arterionephrosclerosis, acute tubular necrosis and diabetic nephropathy which is similar to findings reported by Bradley et al. (Bradley et al., 2020).

Notably, we found that MIA confirmed the cause of death in all 45 cases with a valid, complete set of tissue samples. Additionally, its results made the cause of death more specific in 5 cases (11.11%). In a similar series by D’Onofrio et al. (D'Onofrio et al., 2020) involving 18 subjects, they found MIA to alter the clinical cause of death in 15 cases (83%). However, this series also included other components apart from histopathology including radiological imaging and microbiological examination.

The most common cause of death noted in our series was Type 1 respiratory failure, which correlated with the most commonly affected organ as well as the most common histopathological finding of diffuse alveolar damage seen in 89.79% of the patients. 86.27% of the patients required invasive ventilation prior to their demise, thereby cementing respiratory failure as the most common cause of death. Findings from MIA further helped elaborate the clinical cause of death by explaining the pathology responsible for terminal organ failures observed in our series. The pathological involvement of multiple organs on microscopic analysis, supports our finding that Cov19 infection is a multiorgan process that involves the lung, kidneys, liver, heart and spleen, and may in fact produce pathological changes in other organ systems which were not examined within our study.

MIA cannot detect all clinically relevant findings, a prime example of this would be pulmonary embolism, an important complication in covid-19 infected patients (Klok et al., 2020), for which MIA lacks sensitivity. In large organs, such as the liver and lungs, the locations selected for specimen retrieval may greatly impact eventual findings. For example, the finding of a lung abscess in one of our cases may have been missed entirely had the specimen not been collected from the afflicted site. Therefore, still the conventional autopsy technique remains the gold standard for certifying cause of death because they offer a gross visual survey of entire organ systems. A workaround for this limitation could be the use of radio-imaging to better guide specimen collection (D'Onofrio et al., 2020).

We found the rapid execution of the MIA technique and its inherent minimally invasive nature is more acceptable to the kin of the deceased (Castillo et al., 2016). It theoretically reduces exposure of healthcare workers to virions. Thus, the MIA technique can be a helpful add-on in gaining insights into the cause of death while mitigating risk. A limitation of our study would be the lack of information about microbiological flora, as prevalence of superadded bacterial and fungal infection of the tissues is common. Lack of information of the gross architecture of the organs and organ systems is another factor, which limits the outcomes of the study as stated earlier. There is limited tissue sampling which may not be representative of the pathology. Significant pathology in any other affected organ would likely go unaccounted for other than the ones sampled in the study.

A comparison between the conventional open autopsy technique against the MIA technique would definitely be a more robust way to ascertain the efficacy of MIA. However, we were unable to provide a control arm undergoing open autopsy as the prevalent national guidelines at the time issued by the Indian Council for Medical Research (ICMR) advocated against the use of conventional autopsies for cases that did not have medico-legal implications (Covid19, 2021). One of the strengths of this study is the consecutive enrolment of patients admitted under different specialties irrespective of the severity of SARS CoV19 infection and thereby avoidance of selection bias. Our study shows that use of MIA technique for autopsy can be an acceptable alternative to traditional autopsy. It can reliably confirm or make the cause of death more specific thereby aiding the understanding of the disease progression. However, being a small study with a limited sample size, further trials on a larger scale are warranted. While we employed the technique in a large, tertiary care institute in an Indian metropolis, the technique also holds potential to aid collection of tissue samples in a resource-limited setting (Castillo et al., 2015b).

## Conclusion

Minimally invasive autopsy is a cost-effective tool that can help ascribe and/or augment the probable cause of death in those suffering from SARS-Cov19. It is an easily reproducible technique which not only reduces the time for post-mortem examination, but may reduce the exposure of healthcare workers to potentially infective human tissue. The efficacy of the technique is improved by image-guided targeted tissue biopsies. With the addition of adjuncts such as computed tomography, microbiological assessment of collected tissue samples, the accuracy and diagnostic yield may be enhanced further.

## Data Availability

The datasets used and/or analysed during the current study are available from the corresponding author on reasonable request.

## Abbreviations

SARS CoV19: Severe Acute Respiratory Syndrome Coronavirus 19
WHO: World Health Organisation
MIA: Minimally Invasive Autopsy
CCOD: Clinical Cause of Death
DAD: Diffuse Alveolar Damage
H&E: Hematoxylin and Eosin
ICMR: Indian Council of Medical Research
